# The Relationship Between Cholesterol Level, Cytokine Profile, and Arterial Stiffness in Young Patients with Uncomplicated Type 1 Diabetes

**DOI:** 10.3390/ijms26125513

**Published:** 2025-06-09

**Authors:** Jolanta Neubauer-Geryk, Małgorzata Myśliwiec, Katarzyna Zorena, Leszek Bieniaszewski

**Affiliations:** 1 Clinical Physiology Unit, Medical Simulation Centre, Medical University of Gdańsk, 80-204 Gdańsk, Poland; lbien@gumed.edu.pl; 2 Department of Pediatrics, Diabetology and Endocrinology, Medical University of Gdańsk, 80-211 Gdańsk, Poland; malgorzata.mysliwiec@gumed.edu.pl; 3 Department of Immunobiology and Environment Microbiology, Medical University of Gdańsk, 80-211 Gdańsk, Poland; kzorena@gumed.edu.pl

**Keywords:** arterial stiffness, macrocirculation, pulsatility index, atherosclerosis, cytokines, sVCAM–1, ICAM-1, lipids, type 1 diabetes mellitus, children

## Abstract

Arterial stiffness indicates early atherosclerotic changes prevalent in children and adolescents with type 1 diabetes (T1D), even in those with a well–controlled disease and without additional cardiovascular risk factors. This study aimed to determine whether low–density lipoprotein (LDL) cholesterol and cytokine levels can indicate vascular stiffness in pediatric patients without conventional microangiopathic complications who are not undergoing lipid–lowering therapy. The total study group consisted of 59 pediatric patients divided into two subgroups based on their LDL cholesterol levels and matched for age, age at onset, and duration of diabetes. The investigation involved the precise measurement of several biomarkers including tumor necrosis factor (TNF–α), interleukin 35 (IL-35), interleukin 4 (IL-4), interleukin 10 (IL-10), interleukin 12 (IL-12), interleukin 18 (IL-18), vascular endothelial growth factor (VEGF), Soluble Vascular Cell Adhesion Molecule–1 (sVCAM–1), Intercellular Adhesion Molecule–1 (ICAM-1), Soluble Platelet Selectin (sP–Selectin), Advanced Glycation End Products (AGEs), and Receptors for Advanced Glycation End Products (sRAGE). Arterial stiffness was assessed by calculating pulsatility indices in the common carotid artery and the peripheral arteries in the upper and lower limbs. The comparative analysis indicated that, in the subgroup with LDL cholesterol levels below 100 mg/dL, in comparison to the subgroup with LDL above 100 mg/dL, there was a significant increase in pulsatility indices in elastic and large muscle arteries and notably higher levels of IL-35, IL-10, sVCAM–1, and ICAM-1. This study is the first to recommend the pulsatility index of elastic and large muscular arteries as an effective diagnostic tool for evaluating early atherosclerotic lesions in children and adolescents diagnosed with type 1 diabetes. Elevated LDL cholesterol levels may contribute to vascular stiffness through mechanisms related to a weakened inflammatory response, highlighting the complex interaction between lipid levels, inflammation, and vascular health in patients with type 1 diabetes.

## 1. Introduction

Evidence indicates that accelerated atherosclerosis is present in young people with type 1 diabetes (T1D) compared to a control group without diabetes. The SEARCH study revealed that arterial stiffness, an indicator of early atherosclerotic changes, is prevalent among children and adolescents with T1D and results from poor glycemic control, reduced insulin sensitivity, high BMI (body mass index), uncontrolled blood pressure, and elevated lipid levels. However, the risk of atherosclerotic cardiovascular disease is also elevated in well–controlled T1D patients without additional cardiovascular risk factors, suggesting that other factors may be involved. Chronic hyperglycemia and its consequences are potential contributing factors. The DCCT/EDIC study indicates that glycosylated hemoglobin, lipid, and lipoprotein disorders play significant roles in cardiovascular (CVD) complications. The severity of oxidative stress, vasculitis, and endothelial dysfunction, along with an increased risk of CVD, can also be provoked by hypoglycemia and glycemic variability. Glycemic variability has been shown to stimulate the release of pro–inflammatory cytokines such as IL-6 (interleukin–6) and TNF–α and to induce oxidative stress. While lipid abnormalities in well–controlled T1D patients do not seem to be a substantial contributing factor, statin therapy has been shown to reduce the incidence of ASCVD events in T1D. However, recommendations for diabetic patients under 18 years of age target a low–density lipoprotein (LDL) level of less than 100 mg/dL.

The present study aims to investigate novel associations between LDL cholesterol, inflammatory cytokines, and vascular stiffness in children and young adults with type 1 diabetes who do not exhibit typical microvascular complications and are not taking lipid–lowering drugs. In the studies reported in the literature on vascular stiffness in young patients with diabetes, researchers used such techniques as carotid and femoral artery pulse wave velocity (cfPWV) [1,2,3,4,5,6], ultrafast pulse wave velocity (ufPWV) [7], carotid–radial artery pulse wave velocity, femoral–foot artery pulse wave velocity, and the augmentation index (AIx) [4]. Furthermore, a spectrum of methods based on artery structure was also used: the intima–media thickness (IMT) of the femoral artery [5] and the carotid artery [8,9], including high–frequency ultrasound carotid intima–media thickness (hfCIMT) [7], as well as the carotid artery distensibility coefficient [9] and the flow–mediated dilation (FMD) of the brachial artery [10].

Our study is the first to recommend including the pulsatility index of elastic and large muscular arteries as a diagnostic tool for evaluating early atherosclerotic lesions in children and adolescents with type 1 diabetes. Determining whether these markers can serve as early indicators of cardiovascular risk would facilitate the development of targeted preventive strategies. Identifying these potential early markers may lead to a personalized approach to cardiovascular risk management and prevent adolescent disease progression. This approach emphasizes early risk detection, prevention, and individualized therapy, providing valuable clinical insights and laying the necessary groundwork for future research on timely interventions.

## 2. Results

The study group comprised 59 pediatric patients divided into two subgroups based on their LDL cholesterol level. Subgroup A comprised patients with LDL levels below 100 mg/dL, while subgroup B comprised patients with LDL levels above 100 mg/dL.

### 2.1. Characteristics of Studied Subgroups

The two subgroups did not differ in age, age at onset, or duration of diabetes (see Table 1). The study revealed no statistically significant discrepancies in the distribution of Tanner stages between the subgroups A and B (*p* = 0.16). The subgroup analysis of diabetes patients revealed no significant disparities across BMI, current HbA_1c_ levels, duration of insulin pump therapy, or insulin dosage. Moreover, no substantial variations were observed in the number of episodes of mild and severe hypoglycemia (see Table 1). Subgroups A and B demonstrated no significant disparities in gender distribution or the prevalence of comorbidities, including coeliac disease and Hashimoto’s disease.

### 2.2. Laboratory Examination

Subgroup A, consisting of patients with diabetes, demonstrated a marked lower total cholesterol (A: 148 (125–190) vs. B: 193 (159–288) [mg/dL]; *p* < 0.001) and LDL (A: 89 (61–99) vs. B: 118 (102–188) [mg/dL]; *p* < 0.001) levels. No statistically significant disparities in triglyceride levels or the HDL cholesterol fraction were identified between the two subgroups. However, a significantly higher creatinine concentration was detected in subgroup A with lower LDL cholesterol levels (A: 0.77 (0.5–0.95) vs. B: 0.6 (0.5–0.9) [mg/dL]; *p* = 0.03) (see Table 2). The examined subgroups of diabetic patients exhibited no significant differences in terms of TSH and fT4 concentrations. In addition, no statistically significant differences in creatinine and C–reactive protein concentrations were observed among the subgroups (see Table 2).

### 2.3. Biomarkers Examination

**Figure 1:**
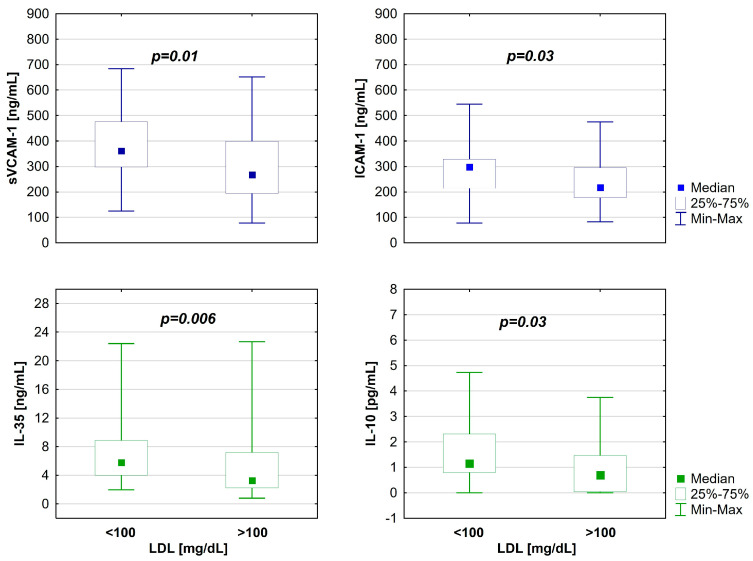
Differences in cytokine levels between the study groups. IL-35—interleukin 35; IL-10—interleukin 10; sVCAM–1—Soluble Vascular Cell Adhesion Molecule–1; ICAM-1—Intercellular Adhesion Molecule–1.

A comparative analysis was conducted on the concentrations of angiogenin, VEGF (vascular endothelial growth factor), and sP-selectin, as well as sRAGE (Receptors for Advanced Glycation End Products) and AGEs (Advanced Glycation End Products) in patients with type 1 diabetes. This study found that the concentrations of these biomarkers were statistically comparable between distinct subgroups of patients with T1D. A comparative analysis of anti–inflammatory cytokine levels among distinct subgroups indicated a substantial increase in IL-35 (interleukin–35) (A: 5.8 (2–22.4) vs. B: 3.3 (0.8–22.7 [ng/mL]; *p* = 0.006) and IL-10 (interleukin–10) (A: 1.2 (0–4.7) vs. B: 0.7 (0–3.8) [pg/mL]; *p* = 0.03) within subgroup A, while IL-4 (interleukin–4) demonstrated no change. No statistically significant differences were observed between this study’s patient subgroups regarding pro-inflammatory cytokines, including TNF–α, IL-12 (interleukin 12), and IL-18 (interleukin-18). However, the investigation revealed that the levels of sVCAM–1 and ICAM-1 were considerably lower in the subgroup of patients with elevated LDL cholesterol levels (267 (76.9–652) vs. 361.5 (124.8–683.7) [pg/mL]; *p* = 0.01 and 217.2 (82.7–475.5) vs. 297 (77.9–544.6) [pg/mL]; *p* = 0.03, respectively) (see Table 3 and Figure 1).

### 2.4. Pulsatility and Blood Pressure Indices

**Figure 2:**
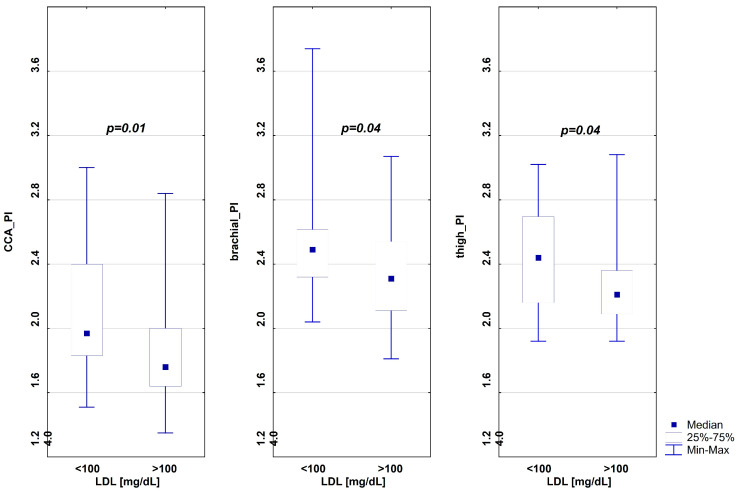
Differences in the pulsatility indices between the study groups. CCA_PI—pulsatility index for common carotid arteries; brachial_PI—pulsatility index for brachial arteries; thigh_PI—pulsatility index for femoral arteries.

A comparison of the examined groups revealed that the carotid pulsatility index (CCA_PI) exhibited significantly higher values in subgroup A. A comparison of pulsatility indices in the examined muscle arteries demonstrated significantly higher pulsatility values at the brachial (brachial_PI) and thigh (thigh_PI) arteries in subgroup A (see Figure 2). No statistically significant differences were observed in other pulsatility indices between the subgroups (Table 4).

### 2.5. Correlations Between Variables

#### 2.5.1. Correlations Between Lipids and Biomarkers

The present study demonstrated that there is a statistically significant negative correlation between the level of LDL cholesterol and the levels of IL-10 (r = −0.29, *p* = 0.03) and IL-35 (r = −0.29, *p* = 0.03), sVCAM–1 (r = −0.31, *p* = 0.02), and ICAM-1 (r = −0.26, *p* = 0.047) (Table 5).

In addition, a significant positive correlation between LDL cholesterol and the TNF-α/IL35 index (r = 0.34, *p* = 0.01) was identified. The present study demonstrated a significant positive correlation between HDL cholesterol and advanced glycation end products (AGEs) (r = 0.29, *p* = 0.03). These significant correlations were exclusively observed in the subgroup with LDL levels above 100. The results of our study demonstrated that both total cholesterol and LDL cholesterol levels were significantly negatively correlated with IL-12 levels (r = −0.44, *p* = 0.009; and r = −0.45, *p* = 0.007, respectively). In addition, a substantial negative correlation was identified between triglyceride levels and sVCAM–1 (r = −0.36, *p* = 0.03) and sP-Selectin (r = −0.34, *p* = 0.048).

#### 2.5.2. Correlations Between Age and Biomarkers

The lack of a statistically significant correlation was found between age, age at onset, duration of diabetes, and the biochemical parameters or biomarkers. Subgroup analysis indicated no statistically significant correlations in the subgroup with LDL levels below 100. In the subgroup with LDL levels greater than 100, a significant negative correlation was found only between age and sVCAM1 (r = −0.43, *p* = 0.009) and between T1D duration and IL-35 (r = −0.40, *p* = 0.02).

#### 2.5.3. Correlations Between Pulsatility Indices, Age, and Lipids

No statistically significant correlation was found between all pulsatility indices and age at onset or triglyceride levels (see Table 6). However, a significant negative correlation was observed between CCA_PI and total cholesterol, as well as LDL and HDL fractions.

Conversely, a substantial positive correlation was identified among the pulsatility indices of the muscular arteries and the subjects’ age. As illustrated in Table 6, some indices were negatively correlated with total cholesterol and LDL levels.

#### 2.5.4. Correlations Between Pulsatility Indices and Biomarkers

This study revealed a substantial positive correlation between the CCA_PI and IL-10 level (r = 0.27, *p* = 0.04), the thigh_PI and IL-12 level (r = 0.27, *p* = 0.04), and also between the brachial_PI and ICAM-1 (r = 0.31, *p* = 0.02).

A correlation analysis examined the relationship between vascular parameters and biomarkers in participants with LDL cholesterol levels exceeding 100 mg/dL. The results revealed significant negative correlations between PI measured above and below the knee or at the ankle and IL-35 or sVCAM–1. This study also found a positive correlation between brachial PI and ICAM-1, and a negative correlation with angiogenin levels. Importantly, CCA_PI showed a positive correlation only with IL-12 levels (see Table 7 for a comprehensive list of significant correlations). In subgroup A, no correlations were detected.

## 3. Discussion

The analysis of the results indicated that, within the subgroup of patients with type 1 diabetes who had an LDL cholesterol level of less than 100 mg/dL, the anti–inflammatory response was significantly more pronounced. These individuals exhibited increased pulsatility, indicative of reduced arterial stiffness in the large elastic and muscular arteries. In contrast, patients with elevated LDL cholesterol levels (higher than 100 mg/dL) showed a lower pulsatility index and a diminished anti–inflammatory response.

To the best of our knowledge, this is the first study of children with uncomplicated type 1 diabetes to examine the relationship between the pulsatility index in multiple elastic and muscular arteries, adhesion molecule concentration, and LDL cholesterol levels.

Atherosclerotic changes in the vessel wall initiate long before the manifestation of symptoms. The earliest intimal changes (fatty streaks) have been documented in the aorta and 50% of right coronary arteries in individuals aged between 15 and 19. The premature emergence of these lesions is influenced by established cardiovascular risk factors, including lipid disorders, smoking, arterial hypertension, BMI, and elevated HbA_1c_ levels [8]. The treatment recommendations for lipid disorders in children with type 1 diabetes indicate that an LDL level of 100 mg/dL should be considered a therapeutic target.

The primary focus of this study is to determine whether elevated pulse wave velocity (PWV), increased IMT, or reduced PI, relative to subjects without diabetes, should be considered as indications for achieving lower LDL levels in the context of hypolipidemic therapy.

It is well established that arterial stiffness serves as a prognostic marker for cardiovascular events [10]. Researchers in this field have primarily used PWV and central blood pressure as crucial indicators of vascular stiffness. In addition, several other factors have been investigated, including the augmentation index, heart rate variability, and FMD.

Dost et al. have posited that elevated pulse pressure in children and adolescents with type 1 diabetes mellitus may serve as an indicator of accelerated vascular stiffness and premature aging [11]. Despite the absence of statistically significant variations in parameters such as blood pressure, heart rate, or pulse pressure among the young T1D patients examined, notable disparities were observed in their pulsatility index.

LDL cholesterol is widely regarded as a pivotal factor in the development of atherosclerotic cardiovascular disease. The importance of LDL–cholesterol–lowering therapy in preventing or slowing atherosclerosis progression has been well established. As demonstrated in [12], the administration of statins during childhood has been shown to retard cIMT thickening and reduce the risk of developing cardiovascular disease in adults.

In addition to the presence of T1D and elevated mean arterial pressure, male sex was identified as the most consistent independent predictor of vascular stiffness [13]. In the present study, the two groups compared did not differ in sex ratio and mean arterial pressure. The Pittsburgh Epidemiology of Diabetes Complications Study demonstrated that LDL cholesterol is a risk factor for cardiovascular disease or major adverse cardiovascular events in type 1 diabetes [14]. A subsequent examination of multiple studies by the Cholesterol Treatment Trialists’ Collaborators revealed that a decrease in LDL cholesterol by 1 mmol/L led to a substantial reduction in the relative risk of major cardiovascular events in patients with T1D, exhibiting a 21% decrease [15]. Therefore, strict control of LDL cholesterol is strongly recommended in patients with T1D. A thorough examination of the extant literature reveals a paucity of studies addressing the modification of lipid levels in children diagnosed with type 1 diabetes. Therefore, data confirming the long–term safety and efficacy of statin treatment on cardiovascular outcomes in children diagnosed with familial hypercholesterolemia are used [12,16].

The Coronary Artery Calcification Study in Type 1 Diabetes (CACTI) demonstrated a correlation between poor glycemic control and abnormalities in the lipid profile. The study revealed a positive association between an increase in HbA_1c_ of 1% and a concomitant rise in LDL cholesterol of 0.1 mmol/L [17]. The findings of our study indicate that, as indicated by HbA_1c_, there is no significant difference in the degree of diabetes control among subgroups that differ in terms of LDL cholesterol levels.

As demonstrated by data from the Swedish National Diabetes Register, age at onset of diabetes is a significant variable in determining the occurrence of cardiovascular complications [18]. A Norwegian study of individuals with type 1 diabetes who had an age of onset before 14 years of age, with an average follow–up period of 24.2 years, demonstrated that the standardized mortality rate due to ischemic heart disease was 20.2/1000 person–years for men and 20.6/1000 person–years for women. In comparison to the general population matched for age and gender, the standardized mortality rate is 4/1000 person–years [19]. However, the subgroups of patients with type 1 diabetes included in our study, which differed in their LDL cholesterol levels, did not demonstrate any differences in age, age at onset, or disease duration.

Endothelial activation represents the initial stage in the development of atherosclerosis, during which adhesion molecules play a pivotal role. Intracellular adhesion molecule–1 (ICAM-1) and vascular cell adhesion molecule–1 (VCAM–1) have been identified as the primary mediators of monocyte and lymphocyte adhesion and migration [19,20]. VCAM–1 has been identified as the most prevalent adhesion molecule in atherosclerosis. It has been demonstrated that VCAM–1 can be released from the surface of the endothelium by proteolytic cleavage to sVCAM–1 [21,22]. There is considerable evidence supporting the utilization of sVCAM–1 as a promising biomarker for atherosclerotic diseases [23]. However, it is important to note that higher levels of sVCAM–1 are also found in type 1 diabetes [24] and other diseases [25,26].

IL-35 is a reactive anti-inflammatory cytokine; its level is increased in various chronic inflammatory diseases, including atherosclerosis [27]. Interleukin-35 has been demonstrated to inhibit the activation of vascular endothelial cells by blocking the expression of VCAM–1 via MAPK–AP1 during acute inflammation induced by lipopolysaccharides. It has been determined that it is a mechanism leading to the inhibition of acute vascular endothelial response [28]. In the present study, elevated levels of anti-inflammatory cytokines, including IL-35, were observed in patients exhibiting elevated levels of soluble adhesion molecules. In experimental studies in mice, Park et al. demonstrated that the tolerance and the reduction in the atherosclerotic burden depend on IL-35 rather than IL-10 [29]. A significant decrease in the levels of circulating IL-35 has been observed in individuals diagnosed with symptomatic coronary atherosclerosis [27]. Moreover, evidence has emerged indicating that IL-35 may contribute to the suppression of proliferative diabetic retinopathy [30]. IL-35 has been demonstrated to possess immunomodulatory properties, which are characterized by the suppression of pro–inflammatory cells and cytokines, the augmentation of IL-10 and transforming growth factor–beta (TGF–beta) secretion, and the stimulation of Treg and Breg [30].

A meta–analysis by Giannopoulou et al. demonstrated that young patients with type 1 diabetes, in comparison to young healthy control subjects, manifest subclinical arterial damage, as indicated by elevated levels of carotid–femoral PWV and cIMT [31]. In a study of young patients with type 1 diabetes, Reis et al. demonstrated that PWV was higher in patients with diabetes than in healthy controls, despite similar levels of pro-inflammatory cytokines in both groups. A negative correlation between IL-10 and lipids was identified in individuals with diabetes [5].

In our previous study, we demonstrated that the presence of autoimmune thyroiditis in children with type 1 diabetes, regardless of lipid levels, impairs elastic artery function as indicated by a decreased pulsatility index [32]. In the present study, negative correlations were identified between IL-10, IL-35, sVCAM 1, ICAM-1, and the level of total and LDL cholesterol (see Table 5). The correlation indicated above was not demonstrated in subgroups categorized based on an LDL cholesterol level of 100 mg/dL. It is important to highlight that the specific pattern of vascular alterations in the distal arteries, which has been observed in diabetic patients, was also identified in a cohort of individuals with LDL levels exceeding 100 mg/dL. These patients are currently classified as having an elevated risk for cardiovascular complications. This group demonstrated a significant negative correlation between pulsatility indices and IL-35, sVCAM-1, and ICAM-1 concentrations (see Table 7).

The present study investigates the differences between subgroups of children with type 1 diabetes who differ in LDL cholesterol levels, higher pulsation index in large elastic and muscular arteries, and higher ICAM-1 or sVCAM–1 levels. We hereby present a potential explanation for the phenomenon that, in patients with LDL lower than 100, higher levels of both IL-35 and sVCAM-1 may manifest as more effective vascular wall protection. This phenomenon is confirmed by the diminished loss of elastic properties, which results from anti-inflammatory and immunoregulatory effects. It is believed that reducing inflammation may play a key role in preventing cardiovascular complications associated with diabetes.

In this study, we observed a significant difference in platelet count between subgroups A and B. Group A, defined by LDL levels below 100 mg/dL, had an average platelet count of 261 (range: 186–387) G/l, while Group B, characterized by LDL levels above 100 mg/dL, exhibited an average platelet count of 298 (range: 151–460) G/l. This difference was statistically significant (*p* = 0.02). A negative correlation was found between the CCA_PI and platelet count (PLT) (r = −0.41; *p* = 0.001) in the study group. In addition, a negative correlation was found between brachial_PI and PLT (r = −0.4; *p* = 0.002). Among patients with elevated LDL levels, a significant negative correlation was observed between CCA_PI and PLT (r = −0.41, *p* = 0.02). In contrast, for those with LDL below 100 mg/dL, a negative correlation was noted between brachial pulse index and PLT (r = −0.42, *p* = 0.04). Research by Di Marco et al. [33] highlighted that, in patients with prediabetes and T2D, platelet reactivity markers and 11–dehydrothromboxane B2 in urine were key determinants of IMT and arterial stiffness parameters. Additionally, a study by Panova-Noeva et al. [34] demonstrated a strong association between platelet volume and augmentation index in the general population. However, the current study does not provide data on platelet function that would permit direct comparisons with the findings reported in the studies mentioned earlier.

In a clinical context, establishing a correlation between these biomarkers and the risk of complications can inform the development of personalized care strategies. These strategies may enable earlier intervention and monitoring of treatment effectiveness. The analysis of cytokines serves as an investigative method that facilitates the identification of simple markers indicative of the early and late stages of atherosclerosis. The evaluation of these factors is facilitated by LDL cholesterol levels, which are generally incorporated into a fundamental health assessment. This study determined that PI does not necessitate specialized equipment, with a Doppler device sufficient for its determination according to the equation presented.

### Limitations

The cross–sectional nature of our study limits the possibility of concluding cause and effect. The absence of studies assessing the properties of large arteries using the pulsatility index in relevant pediatric cohorts precluded the ability to make broad referrals regarding the macrocirculation. Despite the lack of a control group, this is not regarded as a substantial issue, as the main study objective was to compare the outcomes of diabetic patients based on their LDL cholesterol levels. In addition, the limited set of cytokines was due to availability constraints; this study was unable to perform a complete analysis of all potential markers.

The association between Tanner stages and arterial stiffness is attributable to the physiological, hormonal, and growth changes occurring during puberty. Sex hormones such as estradiol and testosterone affect the vessel wall and have the potential to improve arterial elasticity during the later puberty stages [35,36]. Arterial stiffness, measured by PWV, increases with age in children and adolescents. However, the most significant changes in arterial stiffness are typically observed during the pubertal period (Tanner II–V). Research indicates that an increase in arterial stiffness in adolescents with type 1 diabetes may be evident in the earlier stages of Tanner and may worsen with advancing puberty. As outlined in the Results section, we found no significant differences in the distributions of Tanner stages between subgroups A and B. Consequently, we did not use the Tanner staging scale to analyze the studied dependent variables.

It is known that the intensity of physical activity, rather than its duration, plays a key role in preventing premature cardiovascular changes in children with type 1 diabetes. A recent study demonstrated that an increase of 20 min in the amount of daily moderate–to–vigorous physical activity resulted in a decrease in central artery stiffness [37,38]. The variability of the analyzed parameter may be influenced by physical exertion. While we have data concerning the weekly hours of physical activity for most patients, the absence of comprehensive data on the type and intensity of physical activity among all children hinders a thorough assessment.

A randomized controlled trial conducted by Castro-Correia et al. involving children with type 1 diabetes revealed no significant differences in vascular stiffness, as measured by PWV [36], between multiple daily insulin administrations and continuous insulin infusion systems [39]. Furthermore, the analysis of patient subgroups indicated no significant differences among insulin dose units/24 h, insulin dose units per kilogram, and treatment with a pump. Thus, we did not evaluate the impact of the treatment on the analyzed parameters.

## 4. Materials and Methods

### 4.1. Study Design and Population

The present study included 59 patients diagnosed with type 1 diabetes with a mean age of 15.6 ± 1.8 years (boys) and 14.9 ± 2.7 years (girls) (*p* = 0.52). The duration of diabetes was a minimum of 1.2 years.

All patients were treated at the Department of Pediatrics, Diabetology and Endocrinology at the University Clinical Centre in Gdańsk and met the diagnostic criteria for type 1 diabetes according to the International Society for Childhood and Adolescent Diabetes (ISCAD) criteria [40]. As delineated in the previous study [41], the exclusion criteria encompassed micro– and macroangiopathic complications, acute complications of diabetes, the use of lipid–lowering drugs, abnormal thyroid–stimulating hormone (TSH) and free thyroxine concentrations, and systemic diseases such as rheumatoid arthritis and psoriasis. The diagnosis of diabetic retinopathy was excluded based on evidence obtained from fundus examination according to the criteria established by the American Diabetes Association. As delineated in the extant literature, the diagnosis of diabetic neuropathy was made based on subjective and objective neuropathy symptoms. The diabetic nephropathy was determined based on the results of albuminuria tests conducted within the six months before the examination, in addition to the current determination of albumin levels in the collected urine sample. Patients were excluded from the study if they met any of the following criteria: diabetic ketoacidosis, an ongoing infection, uncontrolled celiac disease, or chronic kidney disease at the time of enrolment. Patients were excluded from the study if they had experienced severe hypoglycemia within the previous month. Severe hypoglycemia was defined as a blood glucose level below 54 mg/dL resulting in intervention by another person. Mild hypoglycemia was determined by a blood glucose level of less than 54 mg/dL, which did not necessitate intervention [42]. The participants and their parents were informed of the study’s objectives and methodology, and their consent was subsequently obtained.

The methodology of the study was approved by the Independent Bioethics Committee for Scientific Research at the Medical University of Gdansk (decisions NKBBN/277/2014 of 8 July 2014 and NKBBN/277–512/2016 of 5 December 2016).

### 4.2. Laboratory Analyses

Blood samples were obtained from the participants between 7 and 9 a.m. following an overnight fast. Sera were separated from venous blood within 30 min and stored in a frozen state at −80 °C for up to three months before analysis. All measurements were derived from a single sample.

The following biomarkers were included in the study: HbA_1c_, C–reactive protein (CRP), total cholesterol, high–density lipoprotein cholesterol (HDL–C), low–density lipoprotein cholesterol (LDL–C), triglycerides (TG), thyroid–stimulating hormone (TSH), free triiodothyronine (fT4), serum creatinine, and cytokine levels.

The HbA_1c_ level was determined using immunoturbidimetry utilizing the Unimate 3 kit (Hoffmann–La Roche AG, Basel, Switzerland).

The fasting blood glucose level was determined through an enzymatic test (Roche Diagnostics GmbH, Mannheim, Germany). C–reactive protein level was measured using an immunochemical system (Beckman Instruments, Inc., Galway, Ireland).

Total cholesterol, HDL and LDL cholesterol, and triglyceride levels were measured using Cormay enzyme kits (Cormay, Lublin, Poland).

Serum creatinine levels were measured using the CREA system (Boehringer Mannheim GmbH, Mannheim, Germany).

The serum levels of IL-4, IL-10, and IL-18 were then quantified by ELISA, employing the R&D Systems Quantikine High Sensitivity Human kit (Minneapolis, MN, USA), following the manufacturer’s protocol. The manufacturer set a minimum detectable concentration threshold at 10, 0.5, and 5.15 pg/mL, respectively. The intra– and inter–assay precision of the IL-4, IL-10, and IL-18 tests was determined to be 2.7% and 7.4%, 6.6% and 8.1%, 2.9% and 8.4%, respectively.

The serum concentrations of TNF–α and IL-12 were quantified using an ELISA test (Quantikine High Sensitivity Human from R&D Systems, Minneapolis, MN, USA), following the manufacturer’s instructions. The intra– and inter–assay coefficients of variation (CV) for TNF–α and IL-12 were 6.2% and 2.6%, respectively, and 2.5% and 7.6%, respectively.

The quantification of human IL-35 was conducted using an ELISA assay (Thermo Fisher Scientific, Inc., Waltham, MA, USA). The CV between the tests was determined to be less than 10%, and the CV within was also found to be less than 10%. The sensitivity of the assay was determined to be 9.38 pg/mL.

The concentrations of IL-4, IL-10, IL-18, IL-35, IL-12, and TNF–α were determined by reading at 450 nm on an automated CHROMATE 4300 plate reader (Awareness Technology, Inc., Palm City, FL, USA). The reference curve was generated following the manufacturer’s guidelines.

Serum VEGF levels (VEGF A) were measured using ELISA with the Quantikine High Sensitivity Human kit (R&D Systems, Minneapolis, MN, USA). The assay was performed according to the manufacturer’s instructions catalogued under the code DVE00. The manufacturer established a minimum detectable concentration of 5.0 pg/mL. The coefficient of variation for the internal test was 5.1%, and for the inter–laboratory test, 6.2%.

The serum angiogenin level was measured using an enzyme–linked immunosorbent assay (ELISA) with a commercially available kit (Quantikine High Sensitivity Human from R&D Systems, Minneapolis, MA, USA). This assay has a minimum detectable dose of 0.6 ng/mL, and the intra– and inter–assay coefficients of variation are 3.0% and 8%, respectively.

ICAM-1, sVCAM–1, and sP–selectin concentrations were measured using enzyme–linked immunosorbent assay kits (R&D Systems, Minneapolis, MN, USA). The intra–assay coefficients of variation were 2.8% for sP–selectin, 4.3% for ICAM-1, and 3.3% for sVCAM–1. The lower thresholds for detection in these analytes were set at 1.5 ng/mL for sP–selectin, 5.1 ng/mL for ICAM-1, and 33.8 ng/mL for sVCAM–1. The absorbances of ICAM-1, P–selectin, and sVCAM–1 were measured at a wavelength of 450 nm using an automated CHROMATE 4300 plate reader (Awareness Technology, Inc., Minneapolis, MN, USA). The reference curve was prepared following the specifications outlined by the manufacturer.

Serum AGEs concentrations were measured using an ELISA test (USCN Life Science Inc., Wuhan, China). The assay demonstrated a detection limit of 24 pg/mL, a coefficient of variation between tests of 7.3%, and an intra–assay coefficient of variation of 5.5%.

Serum sRAGE concentrations were determined undiluted using a RAGE sandwich ELISA kit following the manufacturer’s instructions (R&D Systems, Minneapolis, MN, USA). The intra–assay and inter–assay coefficients of variation were 5.9% and 6.7%, respectively. The detection limit of the test was 4.4 pg/mL.

The absorbance was subsequently measured at a wavelength of 450 nm utilizing an automated CHROMATE 4300 plate reader (Awareness Technology, Inc., Minneapolis, MN, USA). The generation of the reference curve followed the manufacturer’s recommendations.

### 4.3. Pulsatility and Blood Pressure Indices

**Figure 3:**
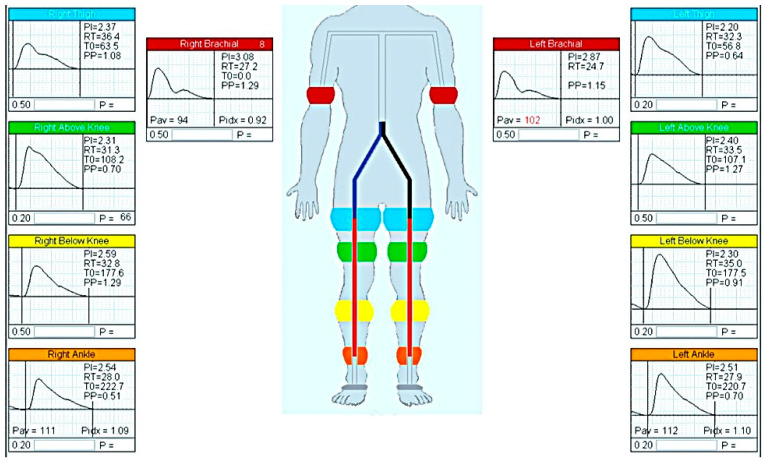
The position of the cuffs during the test is delineated, as are the methods of data and result recording. The image is an original printout of the test. The utilization of color facilitates the selection of appropriate sensors. PI—pulsatility index; RT—rise time; T0—time zero; PP—peak amplitude.

The arterial pulsatility test was conducted in a controlled environment maintained at a constant temperature of approximately 20 °C, as previously described [32,43]. The test was conducted following a 10 min adaptation period in the supine position. The assessment lasted for 30 min. The flow characteristics in the common carotid artery, brachial artery, and arteries of the lower and upper limbs were examined using the VasoGuard 5000 device (Nicoletes, Image Monitoring Inc., Mississauga, ON, Canada). The cuff was placed on the lower limb in four locations: upper thigh, above the knee, below the knee, and above the ankle. The measurements were obtained simultaneously on both sides (see Figure 3). The VasoGuard 5000 software is designed to facilitate the calculation of the pulsatility index for all arteries examined. For further analysis, the mean value of the indices on both sides was calculated.

**Figure 4:**
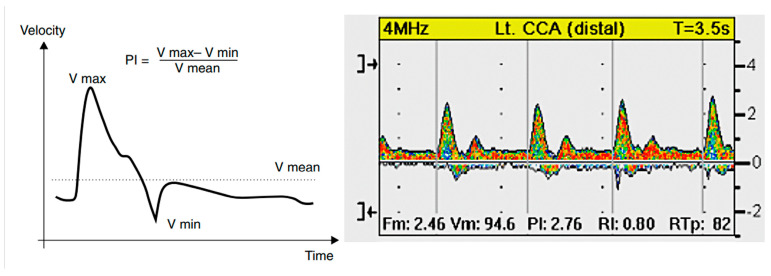
The methodology for calculating the pulsatility index (Gosling Index) (PI). V max—maximal flow velocity, V min—minimal flow velocity, V mean—mean flow velocity (**left side**). The pulse waveform of the common carotid arteries (one of the studied patients) (**right side**).

The measurements on the common carotid artery (Figure 4) were obtained on three occasions on each side. Subsequently, the mean pulsatility index was computed according to Gosling’s formula [31] (see Figure 4).

### 4.4. Statistical Analysis

All data obtained were then subjected to rigorous statistical analysis using the advanced statistical software STATISTICA version 13.1 (StatSoft, Inc., Tulsa, OK, USA), licensed by the CSM GUMed (license no JPZP5077539317AR–H). Initially, the distribution of the variables was assessed using the Shapiro–Wilk test. For variables that exhibited a normal distribution, as indicated by their mean value (SD—standard deviation), the Student’s *t*-test was used. Given the non–normal distribution of the variables, a non–parametric approach was employed, namely the Mann–Whitney U test. The interrelationship of two distinct statistical methodologies, Spearman’s rank correlation and the Chi–square test with Yates’s correction, was used where applicable. The Chi–square test was used to compare gender distributions, distributions of the Tanner scale, and the frequency of hypoglycemic episodes and autoimmune diseases. The study population was selected following the criteria described in Section 4.1. The analysis was conducted without the exclusion of outliers. The statistical significance of the observed results was determined by establishing a significance level of *p* < 0.05.

## 5. Conclusions

The interplay between lipid levels, inflammation, and vascular health in individuals with type 1 diabetes is complex and difficult to unravel. Our study provides valuable insight into this issue.

In patients with uncomplicated type 1 diabetes whose LDL cholesterol levels are above 100 mg/dL, lower cytokine levels are associated with decreased elasticity in large arteries. Therefore, it is reasonable to assume that elevated LDL cholesterol levels may contribute to vascular stiffness through reduced immunological activity.

## Figures and Tables

**Table 1:** Characteristics and comparison of the studied subgroups. Data are presented as median (range)/mean values ± SD.

Characteristics	Diabetic Patients		*p*
	A Group (LDL < 100 mg/dL) n = 24	B Group (LDL ≥ 100 mg/dL) n = 35	
Males, n (%)	12 (50)	15 (42.9)	0.56
BMI [kg/m^2^]	20.4 (16.8–29.6)	20.3 (14.5–26.8)	0.9
Age [years]	16.1 (11.3–18)/16 ± 1.8	14.9 (8.4–18.0)/14.8 ± 2.5	0.1
Onset of diabetes [age]	9.6 (1.8–13.6)/8.5 ± 4.1	7.1 (1.2–12.7)/6.8 ± 3.3	0.1
Diabetes duration [years]	6.9 (1.2–15.9)/7.5 ± 4.6	8.7 (1.7–14.4)/8.1 ± 3.4	0.5
Insulin dose units/24 h	45 (21–70)	45 (20–100)	0.9
Insulin dose units/kg	0.8 (0.4–1.1)	0.8 (0.5–1.4)	0.13
Treatment with pump [%]	50 (0–100)	78 (0–98)	0.45
HbA_1c_ current [%]	7.8 (6.2–11.3)	8.1 (5.9–13.4)	0.3
Episodes of mild hypoglycemia [N/last month]	10 (2–15)	10 (0–30)	0.83
Episodes of severe hypoglycemia [N/last year]	0 (0–1)	0 (0–1)	0.74
Celiac disease, n [%]	2 (8.3)	4 (11.4)	0.99
Autoimmune thyroiditis, n [%]	5 (21)	7 (20)	0.80

The value of *p* < 0.05 was regarded as statistically significant. Abbreviations: HbA_1c_—glycated hemoglobin; BMI—body mass index.

**Table 2:** Comparison of laboratory results in the studied subgroups. Data are presented as the median with the range.

Characteristics	Diabetic Patients		*p*
	A Group (LDL < 100 mg/dL) n = 24	B Group (LDL ≥ 100 mg/dL) n = 35	
CRP [mg/L]	0.3 (0.1–1.3)	0.4 (0.1–4.9)	0.5
Serum creatinine [mg/dL]	0.77 (0.5–0.95)	0.6 (0.5–0.9)	0.03
Creatinine clearance [mL/min]	98 (77–117)	107 (78–147)	0.08
Albuminuria [mg/dL]	7.1 (2.5–88)	6.7 (2.5–27)	0.5
Total cholesterol [mg/dL]	148 (125–190)	193 (159–288)	<0.001
Cholesterol LDL [mg/dL]	89 (61–99)	118 (102–188)	<0.001
Cholesterol HDL [mg/dL]	54 (33–83)	60 (39–90)	0.18
Triglycerides [mg/dL]	69 (34–154)	78 (38–294)	0.24
TSH [mlU/L]	1.9 (0.7–4.2)	1.9 (1–5.1)	0.7
fT4 [pmol/L]	12.9 (10.8–15.0)	12.5 (9–15)	0.3

The value of *p* < 0.05 was regarded as statistically significant. Abbreviations: CRP—C-reactive protein; LDL—low–density lipoproteins; HDL—high–density lipoproteins; TSH—thyroid–stimulating hormone; fT4—free thyroxine.

**Table 3:** Comparison of anti- and pro-inflammatory biomarker results in the studied subgroups. Data are presented as the median with the range.

Characteristics	Diabetic Patients		*p*
	A Group (LDL < 100 mg/dL) n = 24	B Group (LDL ≥ 100 mg/dL) n = 35	
Anti–inflammatory cytokines			
IL-35 [ng/mL]	5.8 (2–22.4)	3.3 (0.8–22.7)	0.006
IL-4 [pg/mL]	5.7 (0–29)	6.4 (0–22.9)	0.59
IL-10 [pg/mL]	1.2 (0–4.7)	0.7 (0–3.8)	0.03
Pro–inflammatory cytokines			
TNF–α [pg/mL]	1.7 (0–6.8)	2.5 (0–5.9)	0.45
IL-12 [pg/mL]	3.0 (0–16.3)	2.5 (0–15.7)	0.85
IL-18 [pg/mL]	65 (34.6–146)	77.2 (44.8–127.8)	0.26
ratio TNF–α/IL-35	0.28 (0–3.44)	0.76 (0–2.97)	0.058
Vascular and inflammatory biomarkers			
Serum angiogenin [ng/mL]	307.5 (112–985)	294.2 (124–799.6)	0.82
VEGF [pg/mL]	232.2 (43–465)	212.8 (55.6–546.2)	0.63
sVCAM–1 [ng/mL]	361.5 (124.8–683.7)	267 (76.9–652)	0.01
ICAM-1 [ng/mL]	297 (77.9–544.6)	217.2 (82.7–475.5)	0.03
sP–Selectin [ng/mL]	293.9 (98.4–731.6)	275 (107–625)	0.61
AGEs [pg/mL]	18,563 (6356–39,420)	19,340 (5480–39,870)	0.78
sRAGE [pg/mL]	1336 (1072.9–2482.9)	1286.8 (788.3–2482.9)	0.65

The value of *p* < 0.05 was regarded as statistically significant. Abbreviations: TNF–α—tumor necrosis factor; IL-35—interleukin 35; IL-4—interleukin 4; IL-10—interleukin 10; IL-12—interleukin 12; IL-18—interleukin 18; ratio TNF–α/IL-35—the ratio of TNF–α and IL-35; VEGF—vascular endothelial growth factor; sVCAM–1—Soluble Vascular Cell Adhesion Molecule–1; ICAM-1—Intercellular Adhesion Molecule–1; sP–Selectin—Soluble Platelet Selectin; AGEs—Advanced Glycation End Products; sRAGE—Receptors for Advanced Glycation End Products.

**Table 4:** Pulsatility and blood pressure indices in the studied subgroups. Data are presented as the median with the range.

Characteristics	Diabetic Patients		*p*
	A Group (LDL < 100 mg/dL) n = 24	B Group (LDL ≥ 100 mg/dL) n = 35	
CCA_PI	2 (1.5–3)	1.8 (1.4–2.8)	0.01
brachial_PI	2.5 (2–3.7)	2.3 (1.8–3.1)	0.04
thigh_PI	2.4 (1.9–3)	2.23 (1.9–3.1)	0.03
above_knee_PI	2.3 (1.8–3.5)	2.2 (1.9–2.8)	0.10
below_knee_PI	2.6 (2–3.6)	2.4 (2.1–3.6)	0.06
ankle_PI	2.5 (2–3.8)	2.4 (2 –3.3)	0.11
Blood pressure and heart rate			
SBP (mmHg)	107 (89–124)	107 (84–126)	1.0
DBP (mmHg)	59 (49–70)	60 (49–76)	0.8
PP (mmHg)	47 (32–70)	47 (34–66)	0.72
HR (beats/minutes)	78 (63–97)	84 (57–111)	0.1

The value of *p* < 0.05 was regarded as statistically significant. Abbreviations: CCA_PI—pulsatility index for common carotid arteries; brachial_PI—pulsatility index for brachial arteries; thigh_PI—pulsatility index for femoral arteries; above_knee_PI—pulsatility index for arteries above knee; below_knee_PI—pulsatility index for arteries below knee; ankle_PI—pulsatility index for arteries at the ankle level; SBP—systolic blood pressure; DBP—diastolic blood pressure; PP—pulse pressure; HR—heart rate.

**Table 5:** The correlation between biomarkers and lipids in the whole study group.

Parameters	Total Cholesterol		Cholesterol LDL		Cholesterol HDL		Triglycerides	
	r	*p*	r	*p*	r	*p*	r	*p*
IL-10	−0.31	0.02	−0.29	0.03	–	ns	–	ns
IL-35	−0.27	0.04	−0.29	0.03	–	ns	–	ns
sVCAM–1	−0.29	0.03	−0.31	0.02	–	ns	–	ns
ICAM-1	–	ns	−0.26	0.047	–	ns	–	ns
AGEs	–	ns	–	ns	0.29	0.03	–	ns

Abbreviations: r—correlation coefficient; ns—non–significant; IL-35—interleukin 35; IL-10—interleukin 10; sVCAM–1—Soluble Vascular Cell Adhesion Molecule–1; ICAM-1—Intercellular Adhesion Molecule–1; AGEs—Advanced Glycation End Products. The correlations among variables were assessed utilizing Spearman’s rank correlation. The value of *p* < 0.05 was regarded as statistically significant.

**Table 6:** The correlation between vascular parameters, lipids, and demographic features in the study group.

Parameters	Age		T1D Onset		T1D Duration		Total Cholesterol		Cholesterol LDL		Cholesterol HDL		Triglycerides	
	r	*p*	r	*p*	r	*p*	r	*p*	r	*p*	r	*p*	r	*p*
CCA_PI	–	ns	–	ns	–	ns	−0.46	<0.001	−0.42	0.001	−0.26	0.04	–	ns
brachial_PI	–	ns	–	ns	–	ns	−0.37	0.004	−0.36	0.005	–	ns	–	ns
thigh_PI	–	ns	–	ns	–	ns	−0.31	0.02	−0.31	0.02	–	ns	–	ns
above_knee_PI	–	ns	–	ns	–	ns	–	ns	–	ns	–	ns	–	ns
below_knee_PI	0.55	<0.001	–	ns	0.27	0.04	−0.31	0.02	−0.31	0.02	–	ns	–	ns
ankle_PI	0.62	<0.001	–	ns	–	ns	–	ns	−0.26	0.046	–	ns	–	ns

Abbreviations: r—correlation coefficient; ns—non significant; CCA_PI—pulsatility index for carotid arteries; brachial_PI—pulsatility index for brachial arteries; thigh_PI—pulsatility index for femoral arteries; above_knee_PI—pulsatility index for muscular arteries above knee; below_knee_PI—pulsatility index for muscular arteries below knee; ankle_PI—pulsatility index for muscular arteries at the ankle level. The correlations among variables were assessed utilizing Spearman’s rank correlation. The value of *p* < 0.05 was regarded as statistically significant.

**Table 7:** The correlation between vascular parameters and biomarkers in the study group and subgroups.

Parameters	IL-35		IL-10		IL-12		sVCAM–1		ICAM-1		Angiogenin	
	r	*p*	r	*p*	r	*p*	r	*p*	r	*p*	r	*p*
Whole study group												
CCA_PI	–	ns	0.27	0.04	–	ns	–	ns	–	ns	–	ns
brachial_PI	–	ns	–	ns	–	ns	–	ns	0.31	0.02	–	ns
thigh_PI	–	ns	–	ns	0.27	0.04	–	ns	–	ns	–	ns
above_knee_PI	–	ns	–	ns	–	ns	–	ns	–	ns	–	ns
below_knee_PI	–	ns	–	ns	–	ns	–	ns	–	ns	–	ns
ankle_PI	–	ns	–	ns	–	ns	–	ns	–	ns	–	ns
Subgroup A												
CCA_PI	–	ns	–	ns	–	ns	–	ns	–	ns	–	ns
brachial_PI	–	ns	–	ns	–	ns	–	ns	–	ns	–	ns
thigh_PI	–	ns	–	ns	–	ns	–	ns	–	ns	–	ns
above_knee_PI	–	ns	–	ns	–	ns	–	ns	–	ns	–	ns
below_knee_PI	–	ns	–	ns	–	ns	–	ns	–	ns	–	ns
ankle_PI	–	ns	–	ns	–	ns	–	ns	–	ns	–	ns
Subgroup B												
CCA_PI	–	ns	–	ns	0.37	0.03	–	ns	–	ns	–	ns
brachial_PI	–	ns	–	ns	–	ns	–	ns	0.42	0.01	−0.36	0.03
thigh_PI	–	ns	–	ns	–	ns	–	ns	–	ns	–	ns
above_knee_PI	−0.41	0.01	–	ns	–	ns	−0.34	0.049	–	ns	–	ns
below_knee_PI	−0.48	0.003	–	ns	–	ns	−0.36	0.03	–	ns	–	ns
ankle_PI	−0.38	0.03	–	ns	–	ns	−0.44	0.009	–	ns	–	ns

Abbreviations: r—correlation coefficient; ns—non–significant; CCA_PI—pulsatility index for carotid artery; brachial_PI—pulsatility index for brachial artery; thigh_PI—pulsatility index for femoral artery; above_knee_PI—pulsatility index for muscular arteries above knee; below_knee_PI—pulsatility index for muscular arteries below knee; ankle_PI—pulsatility index for muscular arteries at the ankle level; IL-35—interleukin 35; IL-10—interleukin 10; IL-12—interleukin 12; sVCAM–1—Soluble Vascular Cell Adhesion Molecule–1; ICAM-1—Intercellular Adhesion Molecule–1. The correlations among variables were assessed utilizing Spearman’s rank correlation. The value of *p* < 0.05 was regarded as statistically significant.

## Data Availability

The data presented in this study are available on request from the corresponding author.

## Abbreviations

The following abbreviations are used in this manuscript:

HbA_1c_—glycated hemoglobin; BMI—body mass index; T1D—diabetes mellitus; CRP–C—reactive protein; LDL—low–density lipoproteins; HDL—high–density lipoproteins; TSH—thyroid–stimulating hormone; fT4—free thyroxine; TNF–α—tumor necrosis factor; IL-35—interleukin 35; IL-4—interleukin 4; IL-10—interleukin 10; IL-12—interleukin 12; IL-18—interleukin 18; ratio TNF–α/IL-35—the ratio of TNF—α and IL-35; VEGF—vascular endothelial growth factor; VCAM–1—Soluble Vascular Cell Adhesion Molecule–1; ICAM-1—Intercellular Adhesion Molecule–1; sP–Selectin—Soluble Platelet Selectin; AGEs—Advanced Glycation End Products; sRAGE—Receptors for Advanced Glycation End Products; CCA_PI—pulsatility index for common carotid arteries; thigh_PI—pulsatility index for femoral arteries; above_knee_PI—pulsatility index for arteries above knee; below_knee_PI—pulsatility index for arteries below knee; ankle_PI—pulsatility index for arteries at the ankle level; SBP—systolic blood pressure; DBP—diastolic blood pressure; PP—pulse pressure; HR—heart rate; PWV—pulse wave velocity; IMT—intima–media thickness; FMD—flow–mediated dilation.
